# Acute Disseminated Encephalomyelitis Onset: Evaluation Based on Vaccine Adverse Events Reporting Systems

**DOI:** 10.1371/journal.pone.0077766

**Published:** 2013-10-16

**Authors:** Paolo Pellegrino, Carla Carnovale, Valentina Perrone, Marco Pozzi, Stefania Antoniazzi, Emilio Clementi, Sonia Radice

**Affiliations:** 1 Unit of Clinical Pharmacology, University Hospital “Luigi Sacco”, Università di Milano, Milan, Italy; 2 Scientific Institute IRCCS E. Medea, 23842 Bosisio Parini, Lecco, Italy; 3 IRCCS Foundation Ca’ Granda Ospedale Maggiore Policlinico, Milan, Italy; University Hospital Basel, Switzerland

## Abstract

**Objective:**

To evaluate epidemiological features of post vaccine acute disseminated encephalomyelitis (ADEM) by considering data from different pharmacovigilance surveillance systems.

**Methods:**

The Vaccine Adverse Event Reporting System (VAERS) database and the EudraVigilance post-authorisation module (EVPM) were searched to identify post vaccine ADEM cases. Epidemiological features including sex and related vaccines were analysed.

**Results:**

We retrieved 205 and 236 ADEM cases from the EVPM and VAERS databases, respectively, of which 404 were considered for epidemiological analysis following verification and causality assessment. Half of the patients had less than 18 years and with a slight male predominance. The time interval from vaccination to ADEM onset was 2-30 days in 61% of the cases. Vaccine against seasonal flu and human papilloma virus vaccine were those most frequently associated with ADEM, accounting for almost 30% of the total cases. Mean number of reports per year between 2005 and 2012 in VAERS database was 40±21.7, decreasing after 2010 mainly because of a reduction of reports associated with human papilloma virus and Diphtheria, Pertussis, Tetanus, Polio and *Haemophilus* Influentiae type B vaccines.

**Conclusions:**

This study has a high epidemiological power as it is based on information on adverse events having occurred in over one billion people. It suffers from lack of rigorous case verification due to the weakness intrinsic to the surveillance databases used. At variance with previous reports on a prevalence of ADEM in childhood we demonstrate that it may occur at any age when post vaccination. This study also shows that the diminishing trend in post vaccine ADEM reporting related to Diphtheria, Pertussis, Tetanus, Polio and *Haemophilus* Influentiae type B and human papilloma virus vaccine groups is most likely due to a decline in vaccine coverage indicative of a reduced attention to this adverse drug reaction.

## Introduction

Acute disseminated encephalomyelitis (ADEM) is an immune mediated inflammatory disorder of the central nervous system (CNS) that commonly occurs within one month from antigenic challenge [1]. Despite the majority of ADEM cases being attributed to a post-infectious aetiology, other causes were reported [2]. Post vaccine aetiology was described for 5% of all ADEM cases [2] and several vaccines have been described to be related to this condition [2,3]. The incidence of ADEM onset ranges from 1/10^6^ to 1/10^5^ and may change between different vaccine formulations [3]. Epidemiological data about this adverse event are still missing; this may be due to the rarity of post vaccine ADEM.

A possibility to tackle this issue is to rely on information from pharmacovigilance (PV) databases. Several PV programmes have aimed at identifying the onset of adverse reactions to drugs and vaccines. These programmes are particularly valuable as they collect spontaneous reports on large populations. 

One of the biggest national and international databases is the Vaccine Adverse Event Reporting System (VAERS). VAERS collects approximately 28,000 adverse drug reactions (ADRs) following vaccination per year [4]. Despite its known limitations, this passive system allows identification of rare and severe adverse events following vaccination [5].

Another international database, the EudraVigilance post-authorisation module (EVPM) is a database of all reports of suspected serious adverse reactions from the European Union (EU) [6]. As a whole these databases retrieve ADR from more than one billion people. 

The goal of this study was to evaluate epidemiological features of post vaccine ADEM considering both the EVPM and VAERS databases. 

## Materials and Methods

### Case selection

For this surveillance, we considered both the EVPM and the VAERS database for national (domestic) and international (non-domestic) ADRs. From VAERS we were able to extract data regarding age, gender, onset interval, clinical outcome as well as related vaccine and year of vaccination. For each reports case description was extracted and considered for case evaluation.

Data from the EVPM database were instead extracted though manual search among ADR reports organised by substances. For each vaccine, we extracted data regarding age, gender and clinical outcome. Onset interval and year of vaccination were not available. 

### Case evaluation

Cases retrieved from the VAERS database were reviewed considering the signs, symptoms and diagnosis within each identified report. A case was considered verified if: (i) the report clearly stated the diagnosis of ADEM or described signs and symptoms consistent with ADEM diagnosis [1]; (ii) the patient was not diagnosed with Multiple Sclerosis; (iii) the report did not describe any previous infective episode. Unverified cases were excluded from further analyses. 

### Causality evaluation

Causality evaluation of VAERS cases was performed using the WHO criteria [7] in order to minimise the known limits of these databases, i.e. misreporting and low data quality. Almost 30% of ADEM onset do not relate to a clinically evident infection or vaccination [1]. The classification term “very likely/certain” was therefore not used in this classification since it requires that post vaccination ADEM “could not be explained by current disease”. Cases defined as “unrelated” or “unknown” were excluded from further analyses.

We did not carry out causality evaluation of EVPM cases as this information is already provided as such by the database itself. 

Two different referees with comparable PV expertise evaluated each report and supplementary materials in order to classify the causal association between vaccination and ADEM using WHO criteria. The referees completed their causality assessment independently and they resolved any discordance via internal discussion. 

### Epidemiological analysis

A descriptive statistical analysis was performed to summarise the main characteristics of the cases retrieved from the VAERS and EVPM databases. Due to the differences among these databases, we analysed and present data distinct by database. 

For each database the distribution of the related vaccines was analysed and described. 

Frequency of ADEM was assessed by considering VAERS data from the time frame between 2005 and 2012. Data from year 2013 were not included in the analysis to prevent any possible bias. A descriptive analysis of related vaccines was performed considering the overall and per year frequencies. 

In order to evaluate the causes of variability in related vaccines within the per year frequency of ADEM cases, data regarding vaccine coverage in the US [8] with relative cases was used as a comparison. 

All statistical analyses were performed with the MedCalc v.12.1.4 software (BVBA).

## Results

### Case selection and causality assessment

From the VAERS database we retrieved 236 cases of ADEM following vaccination. 

Causality assessment, performed using the WHO criteria, showed a probable relationship between vaccine and ADEM in 38% of the cases, while only 9% (20 cases) were unrelated (table 1). 15% was found to be “unrelated”, “unclassifiable” or excluded because not verified during initial assessment while 85% of the cases was classified as ”probable”, “possible” or “unlikely”. These 199 cases were considered for the epidemiological analysis. Likewise, we retrieved 205 cases from the EVPM database that were classified as related to vaccination and thus used in the epidemiological analysis.

**Table 1:** Demographic data from surveillance databases.

Characteristic		VAERS (%)	EVPM (%)
Age			
	0-17 years	99 (48%)	94(48%)
	18-64	79 (40%)	92 (45%)
	65+ years	12 (6%)	7 (3%)
	Unknown/not reported	14 (7%)	7 (3%)
Sex			
	Male	93 (47%)	80 (39%)
	Female	101 (51%)	123 (60%)
	Unknown/not reported	5 (3%)	2 (1%)
Interval from vaccination to ADEM onset (days)			
	0-2 days	41 (21%)	
	2-30 days	121 (61%)	
	More than 30 days	37 (19%)	
Causality assessment			
	Probable	90 (38%)	
	Possible	77 (33%)	
	Unlikely	32 (14%)	
	Unrelated, unknown, unverified	37 (15%)	

### Patients’ characteristics

Among the VAERS reports considered in our analysis, 93% included information on patients’ age and 97% on patients’ gender (table 1). Almost half of the patients had less than 18 years, while only 6% were over 65. We observed a similar age distribution among the EVPM reports, with seven cases (3%) older than 65 years and 99 cases (48%) younger than 18 years (table 1).

Among the VAERS reports there was a slight predominance of females (table 1), as 12% of the reports was related to the sex specific vaccination against human papilloma virus (HPV). Consistent with the VAERS data, reports from the EVPM database also showed a predominance of female patients mainly due to sex specific vaccination (table 1). Correcting for this factor revealed a slight male predominance, in both databases with a female/male ratio of 0.66 and 0.91 for the VAERS and EVPM databases, respectively.

All reports from the VAERS database recorded the time elapsed between vaccination and ADEM symptoms onset (table 1). The time interval ranged between 2-30 days from vaccination in 61% of the cases, while 37 patients (19%) developed ADEM after one month. Such information is not available on the EVPM database.

### Related vaccines

**Figure 1:**
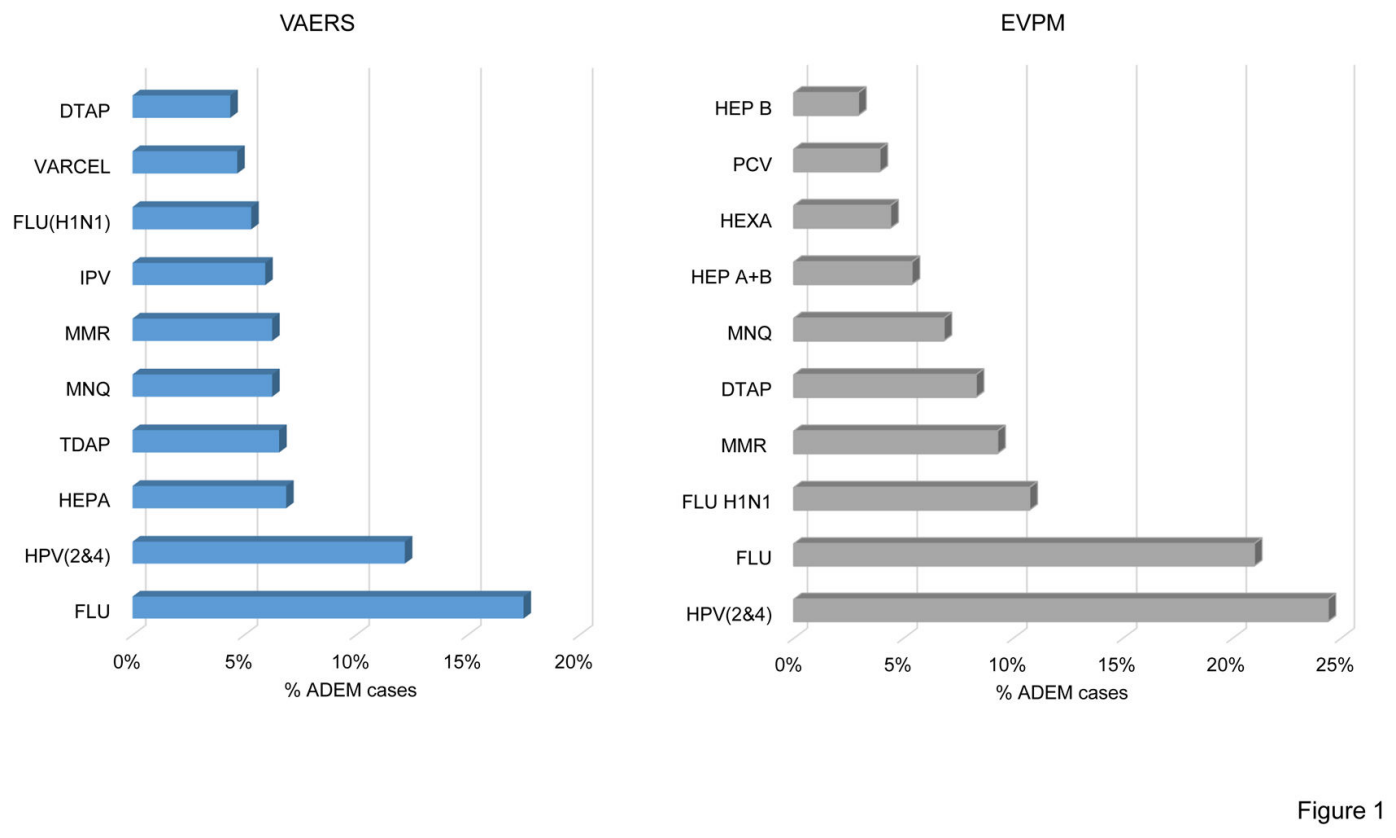
Most commonly related vaccine in the VAERS and EVPM databases. The histograms show the relative distribution of the ten commonest vaccine related to ADEM cases in the VAERS and EVPM databases. HPV (2&4): human papilloma virus vaccine, bivalent and quadrivalent; FLU: seasonal flu vaccine; FLU H1N1: H1N1 vaccine; MMR: measles, mumps, and rubella vaccine; PCV: pneumococcal conjugate vaccine; MNQ meningococcal vaccine; DTAP: Diphtheria, Pertussis, Tetanus; HEP B: Hepatises B vaccine; HEP A+B: Hepatitis A and B vaccine; HEXA: hexavalent vaccines; VARCEL: varicella vaccine; IPV: inactivated poliovirus vaccine; TDAP: vaccine against tetanus, diphtheria, and pertussis in adolescents.

Vaccination against seasonal flu (FLU) and HPV (HPV2&4) were those most frequently associated with ADEM development in both the VAERS and EVPM databases, accounting together for almost 35% of the overall reports (Figure 1). 

The proportion of ADEM cases following the ten most frequently reported vaccine was 76% in the VAERS database and 97% in the EVPM database (Figure 1). 

By grouping together vaccinations against Diphtheria, Pertussis, Tetanus, Polio and *Haemophilus* Influentiae type B (DTaP+IPV+HiB), we found that this was the vaccine group most frequently associated with ADEM development in the VAERS database (21%). This vaccine group accounted instead for less than 15% of the ADEM cases in the EVPM database. 

**Figure 2:**
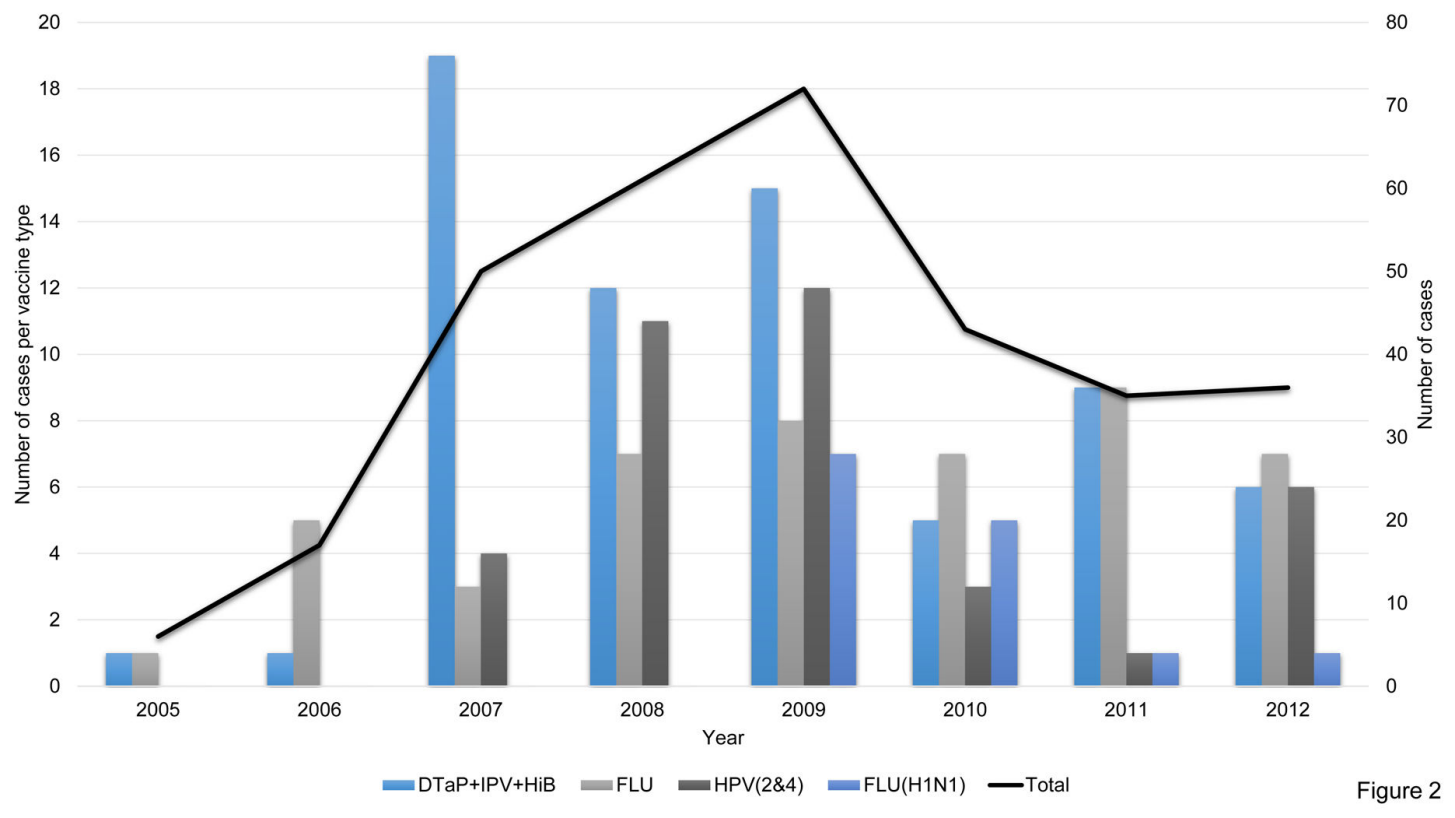
ADEM cases from the VAERS database. The graph shows the number of cases (black line) and the frequency of specific vaccine groups per years. DTaP+IPV+HiB: Vaccine against Diphtheria, Pertussis, Tetanus, Polio and *Haemophilus* Influentiae type B; FLU: seasonal flu vaccine; FLU H1N1: H1N1 vaccine; HPV (2&4): human papilloma virus vaccine, bivalent and quadrivalent.

Figure 2 shows the frequency of post vaccine ADEM assessed between 2005 and 2012 in the VAERS database; the mean number of reports per year was 40±21.7, with the highest number of reports in 2009. Since 2010, the frequency of ADEM reporting per year decreased mainly due to a reduction of reports associated with HPV2&4 and DTaP+IPV+HiB vaccines (Figure 2). The frequency of ADEM reports related to flu vaccination was instead constant in this period. 

We observed a discrepancy between the decreasing frequencies of ADEM reports per year following HPV2&4 and DTaP+IPV+HiB vaccines and vaccine coverage in the same time-span which was constant (DTaP+IPV+HiB) or increased (HPV2&4). This analysis was not done on the EVPM database as it does not report the year of ADEM onset.

**Figure 3:**
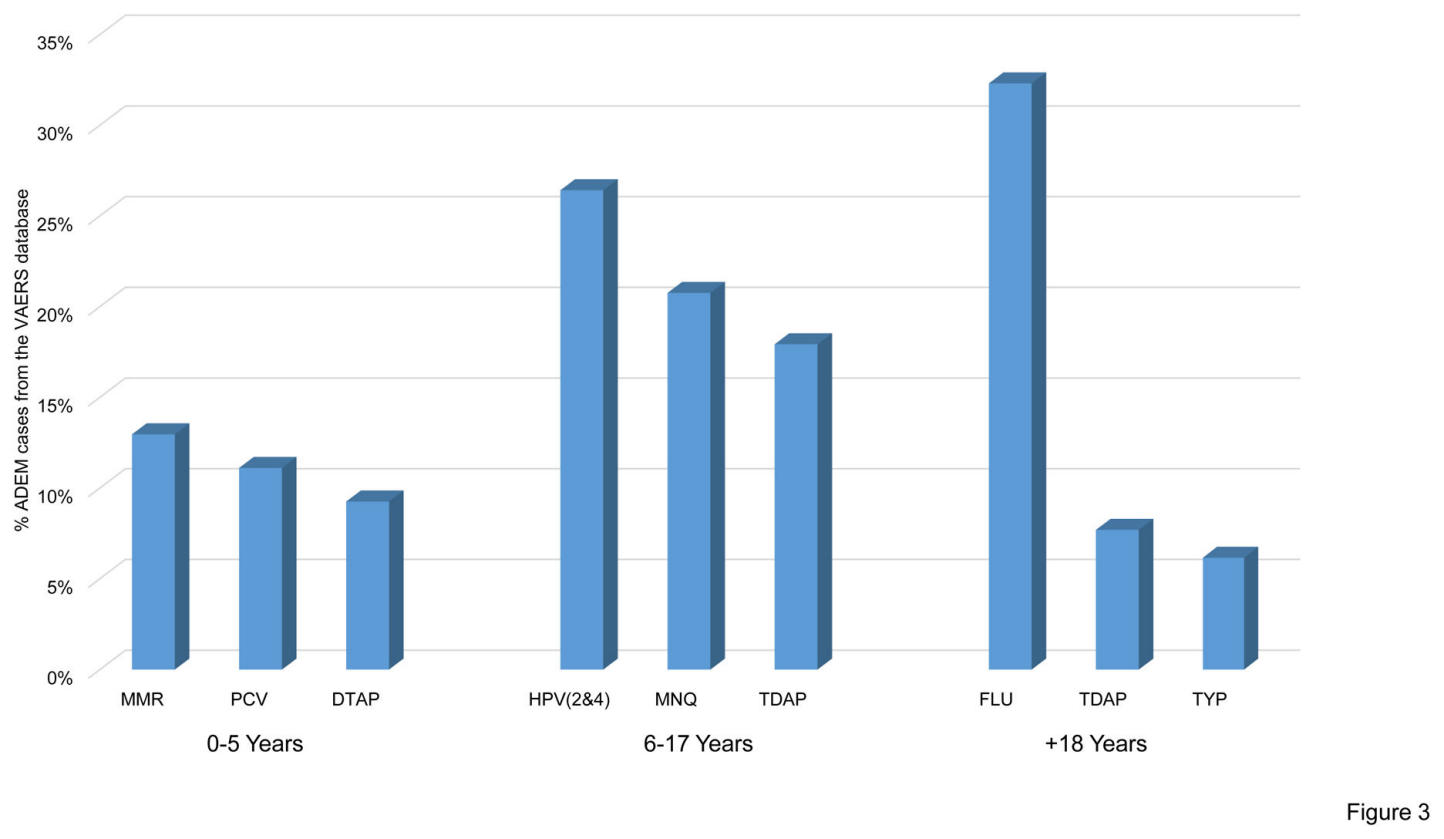
Most frequently reported vaccines in the VAERS database divided by age groups. The histogram shows the relative distribution of the three most common vaccine related to ADEM cases in the VAERS database by considering different age groups. MMR: measles, mumps, and rubella vaccine; PCV: pneumococcal conjugate vaccine; DTAP: Diphtheria, Pertussis, Tetanus vaccine; HPV (2&4): human papilloma virus vaccine, bivalent and quadrivalent; MNQ meningococcal vaccine; TDAP: vaccine against tetanus, diphtheria, and pertussis in adolescents; FLU: seasonal flu vaccine; TYP: Typhoid Vaccine.

**Figure 4:**
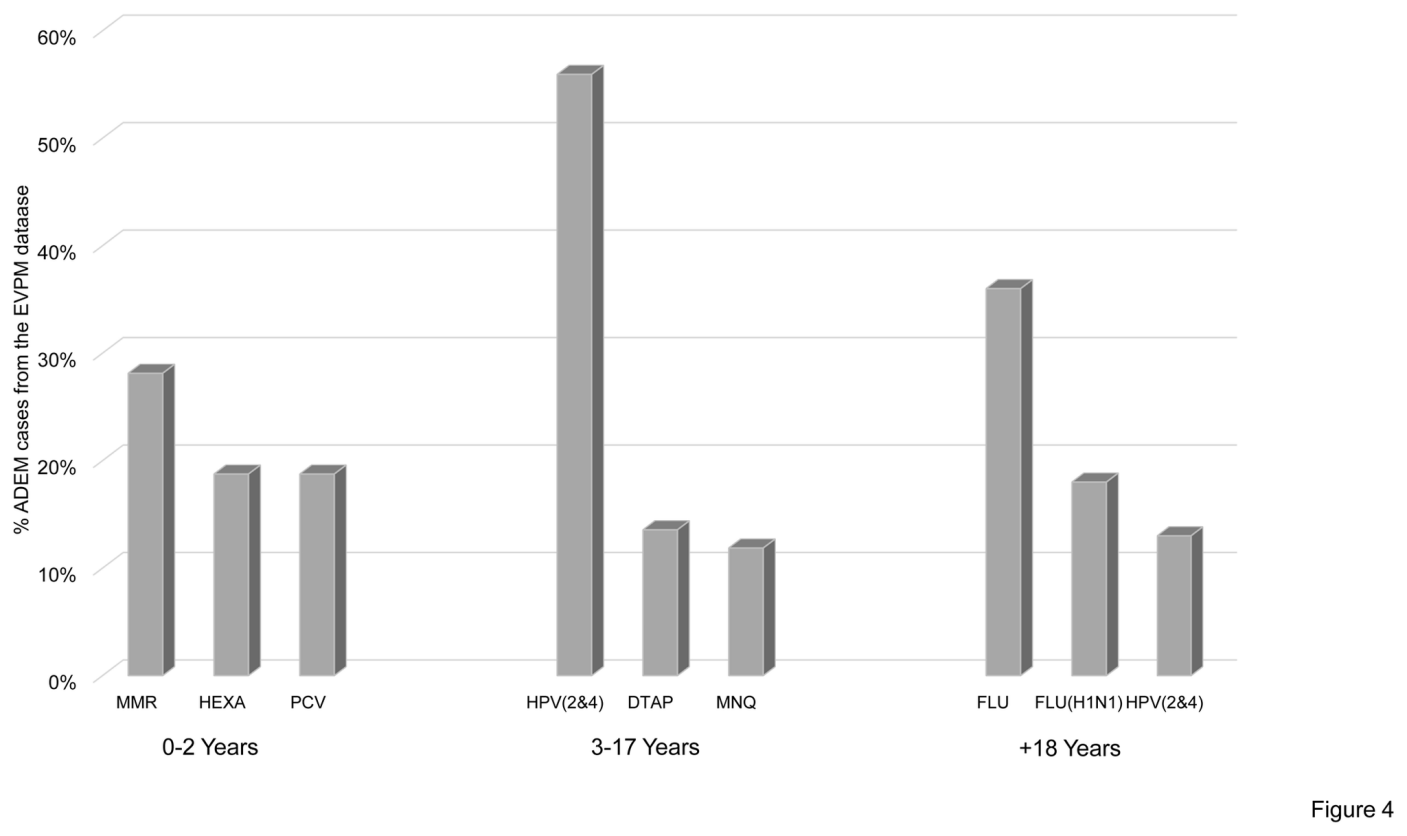
Most frequently reported vaccines in the EVPM database divided by age groups. The histogram shows the relative distribution of the three commonest vaccines related to ADEM cases in the EVPM database by considering different age groups. MMR: measles, mumps, and rubella vaccine; PCV: pneumococcal conjugate vaccine; DTAP: Vaccine against Diphtheria, Pertussis, Tetanus; HPV (2&4): human papilloma virus vaccine, bivalent and quadrivalent; MNQ meningococcal vaccine; HEXA: hexavalent vaccines; FLU: seasonal flu vaccine; FLU H1N1: H1N1 vaccine.

By considering vaccines most commonly involved in a specific age group from the VAERS database, we observed that vaccines against measles, mumps, and rubella (MMR) and FLU were those most commonly involved in the 0-5 years age group (13%), followed by pneumococcal conjugate vaccine (PCV) (11%) and DTaP (9%). Vaccines against HPV(2&4) and meningococcus (MNQ) represented the most frequently suspected causes of ADEM in the age group 6-17 years with 26% and 21% of the cases, respectively. Seasonal flu vaccine was the most frequently suspected cause of ADEM after 18 years, representing 32% of the total cases (Figure 3). Data from the EVPM database were consistent with those of the VAERS database (Figure 4). 

## Discussion

In this study we report the first characterisation of epidemiologic data regarding ADEM following vaccination considering a large number of reports arising from the two largest PV safety databases, covering more than 1 billion persons in the US and EU. 

Previous reports on ADEM indicated that this condition is commoner in children than in adults and is characterised by a mean age of onset ranging from 5 to 8 years [1]. By contrast, our study clearly shows that ADEM following vaccination occurred in patients older than 18 years in almost 40% of the cases. This suggest that ADEM, at least when post vaccine, is not restricted to a specific age and that previously reported age-related associations are linked to a specific setting in which the observations were made [1,2]. 

Consistent with this hypothesis we observed a shift in suspected vaccines in different age groups: vaccine against PCV, DTaP and MMR represented the most frequently suspected group of ADEM occurring between 0 and 5 years while ADEM following vaccination against HPV were the most frequently reported in the age group 6-17 years. After 18 years, FLU represented the vaccination most frequently associated with ADEM. A male predominance for ADEM had been described in two paediatric cohorts [9,10], though globally no gender predominance was reported. Consistent with these two studies and after correction for the presence of female patients with ADEM following HPV2&4 vaccine, we observed a male predominance in post vaccination ADEM.

Time between antigenic challenge and ADEM onset is generally described as ranging between 2 days and 4 weeks [1] even though this varies among different studies and almost 25% of the patients did not report any antecedent infection or vaccination within a few weeks before ADEM onset [1]. According to these data, our study shows that ADEM developed after 30 days from vaccination in only 19% of cases. From the information available to us we cannot determine whether this delayed onset underlies a delayed onset of this ADR or is due to unreported or subclinical/undetected infection having occurred in the period between vaccination and the onset of ADEM.

The highest frequency of ADEM observed following FLU and HPV(2&4) does not automatically imply that these vaccines are associated with a higher risk of ADEM development. Vaccination against HPV was introduced only recently for small target populations and this may increase awareness of the physicians to subsequent ADRs resulting in a high number of reported cases. On the contrary, vaccination against FLU is administered to a larger number of subjects thus high numbers of reports may be the consequence of the large distribution of the vaccine despite a lower incidence of ADEM following it [11,12]. Other factors, such as the limited interest into an already know ADR following FLU vaccine, could also affect reporting rate, resulting in a lower incidence. 

It was previously stated that the vaccinations against MMR are those most commonly associated with ADEM [13]. By contrast, our data, obtained from a large database analysis, indicate that this is not the case as only a small amount of total ADEM cases was related to this group of vaccines with respect of the large number of doses distributed and high vaccine coverage in US and EU [14]. 

Since these data arise from passive surveillances systems, we cannot draw conclusions on the possible differences in the risk of ADEM following a specific vaccine. 

This study shows a diminishing trend in post vaccine ADEM reporting related to the DTaP+IPV+HiB vaccine group, which was frequently reported between 2007 and 2009 but less represented in the following years. Yet the coverage of this vaccine group among the US population has remained fairly constant [8]. We found a similar trend also for the vaccination against HPV with at least one dose. This vaccination increased from 25% in 2007 to 51% in 2011 [8], yet the frequency of reports due to HPV vaccine decreased. Such discrepancies in vaccination coverage *vs.* ADEM reports may indicate a lower interest on ADRs, which is a known cause of under-reporting [5]. 

This study has some limitations as it is a retrospective study based on data retrieved from passive PV surveillance systems, which may suffer from under-reporting and differential reporting [15]. An important issue of these epidemiological studies is the lack of a rigorous verification of the cases and the different way of data verification in the two databases.

Since the VAERS staff follows up on all serious ADR to obtain additional medical records, many of the ADEM reports are analysed further and the risk of misdiagnosis thus reduced. 

Reports to EVPM database arise from medical doctors or pharmacists who directly interact with the patient. With this system, the number of unchecked reports is very low and the quality of reports should be higher. 

Despite these mechanisms of verification and our performing case verification, the risk of misdiagnosis that could possibly lead to the introduction of false positive and the exclusion of false negative cases in this analysis has to be highlighted. 

Another possible issue that should be highlighted is the risk of case overlaps between the EVPM and VAERS databases limited to the VAERS non domestic cases since we cannot evaluate the geographical source of each of them. 

Since the EVPM and VAERS databases do not provide information on patient race or ethnic group, we were unable to analyse such factors. Further studies based on different databases may provide insight in this direction. 

Despite these limitations, we provide the first epidemiological study analysing the onset of post vaccine ADEM with special regards to age of onset, sex and related vaccines. Our findings do not support previous evidence in the literature on an increased frequency of ADEM following vaccination in childhood and highlight the role of the different vaccines during lifetime. Moreover, we observed a shift in the ADEM-related vaccines that may be explained by a change in the reporting pattern, rather than by a change in ADEM epidemiology. 

Higher attention towards this specific ADR would be important in order to define better the incidence of post vaccine ADEM. 
