# Correction: Acute Disseminated Encephalomyelitis Onset: Evaluation Based on Vaccine Adverse Events Reporting Systems

**DOI:** 10.1371/annotation/1d544202-04f5-4848-83f1-696c2de4221e

**Published:** 2013-12-30

**Authors:** Paolo Pellegrino, Carla Carnovale, Valentina Perrone, Marco Pozzi, Stefania Antoniazzi, Emilio Clementi, Sonia Radice

In the last sentence of the abstract the word “not” has been inadvertently omitted between the words "likely" and "due", thus making the sentence convey a message opposite to the one it should have conveyed. The publisher apologizes for this typesetting error.

